# A miniaturized mechanical antenna based on FEP/THV unipolar electrets for extremely low frequency transmission

**DOI:** 10.1038/s41378-022-00395-x

**Published:** 2022-05-31

**Authors:** Yong Cui, Ming Wu, Zhaoyang Li, Xiao Song, Chen Wang, Haiwen Yuan, Zhi-Xin Yang, Junwen Zhong

**Affiliations:** 1 School of Automation Science and Electrical Engineering, Beihang University, Beijing, 100191 China; 2 Department of Electromechanical Engineering and Centre for Artificial Intelligence and Robotics, University of Macau, Macau, SAR 999078 China; 3 School of Cyber Science and Technology, Beihang university, Beijing, 100191 China; 4 State Key Laboratory of Internet of Things for Smart City and Department of Electromechanical Engineering, University of Macau, Macau, SAR 999078 China

**Keywords:** Electrical and electronic engineering, Electronic properties and materials

## Abstract

An electret-based mechanical antenna (EBMA), which can transmit extremely low frequency (ELF) electromagnetic signals, has the advantages of miniaturization and high transmitting efficiency, with great potential applications in air, underwater, and underground communications. To improve the charge density of the electret, which is a key factor in determining the radiation performance of an EBMA, this work proposes a fluorinated ethylene propylene/terpolymer of tetrafluoroethylene, hexafluoropropylene and vinylidene fluoride (FEP/THV) unipolar electret exhibiting negative polarity, reaching a total charge density up to −0.46 mC/m^2^ for each layer of electret. Long transmission distances can be achieved in sea water, soil, and air using a 3-layer-FEP/THV-based EBMA with a compact volume of 5 × 10^−4^ m^3^. As an application demonstration, binary ASCII-coded ELF information of “BUAA” is successfully transmitted with a power consumption < 5 W.

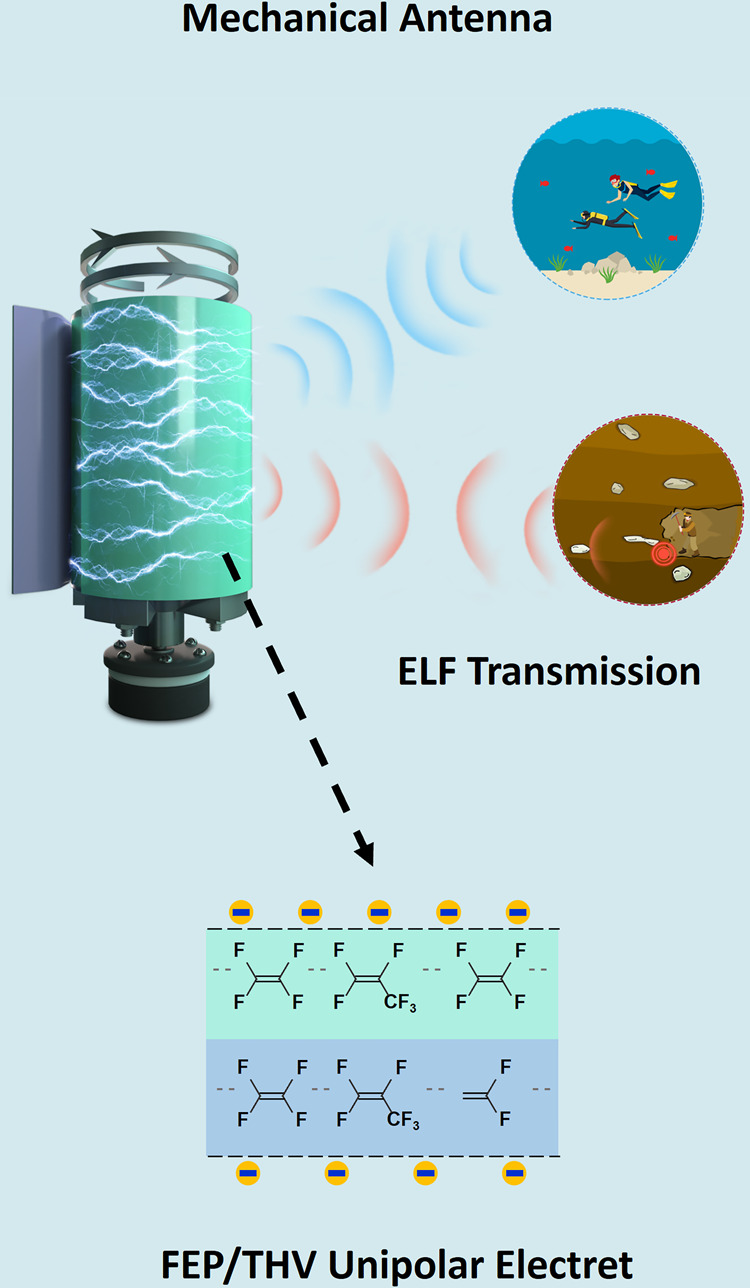

## Introduction

Extremely low frequency (ELF, 3–30 Hz) electromagnetic waves have low attenuation and strong penetration in transmitting media. These characteristics make ELF electromagnetic waves suitable for air, underwater, and underground communications. However, traditional electromagnetism ELF transmitting antennas with a basic working mechanism of resonance current have drawbacks of large size, high power consumption, and low transmitting efficiency^1–4^. In recent years, mechanical antennas have been proposed to address the existing issues of ELF transmitters. To generate electromagnetic radiation with a mechanical antenna, cyclic mechanical motion is applied to drive the net electrostatic charges, electrical dipoles, or magnetic dipoles^5–9^. The current distribution and electromagnetic energy of a mechanical antenna are independent of the power supply and more controllable than those of a traditional antenna. This unique radiation mechanism makes the mechanical antenna free from the limitation of antenna size in terms of efficiency, thus achieving high-efficiency ELF electromagnetic radiation with a size much smaller than that of a traditional ELF transmitting antenna^10–13^.

Compared to mechanical antennas based on permanent magnets (magnetic dipoles) or piezoelectric materials (electrical dipoles), electret-based mechanical antennas (EBMAs) have higher radiation efficiency and are more advantageous in long-distance radiation^14–16^. Specifically, electrets are used as carriers of net electrostatic charges. Then, the electret is driven by a controllable mechanical device to achieve ELF transmission^17–20^. As a result, the total charge density characterizing the amount of net charge carried by the electret is a key factor in determining the antenna’s radiation intensity. A traditional EBMA is constructed with either bipolar electrets or unipolar electrets with one metallized surface. These two types of electrets are not the most suitable electrets for EBMAs, and their charge distribution limits the performance of EBMAs. For bipolar electrets, there are charges of opposite polarity stored on the two surfaces of the electret, and the electromagnetic field generated by the movement of the positive and negative charges will cancel each other. Therefore, only the electromagnetic radiation produced by the difference between the positive and negative charges is effective. Electrets with one metallized surface belonging to unipolar electrets carry net charges with one polarity^21–25^, which are more suitable for application in EBMAs than bipolar electrets. However, the metallized surface is a redundant structure, and only one surface of these electrets carries charges, which is not helpful for improving the miniaturization and transmitting efficiency of an EBMA^26^. A new strategy needs to be proposed to further enhance the performance of EBMAs.

**Fig. 1:**
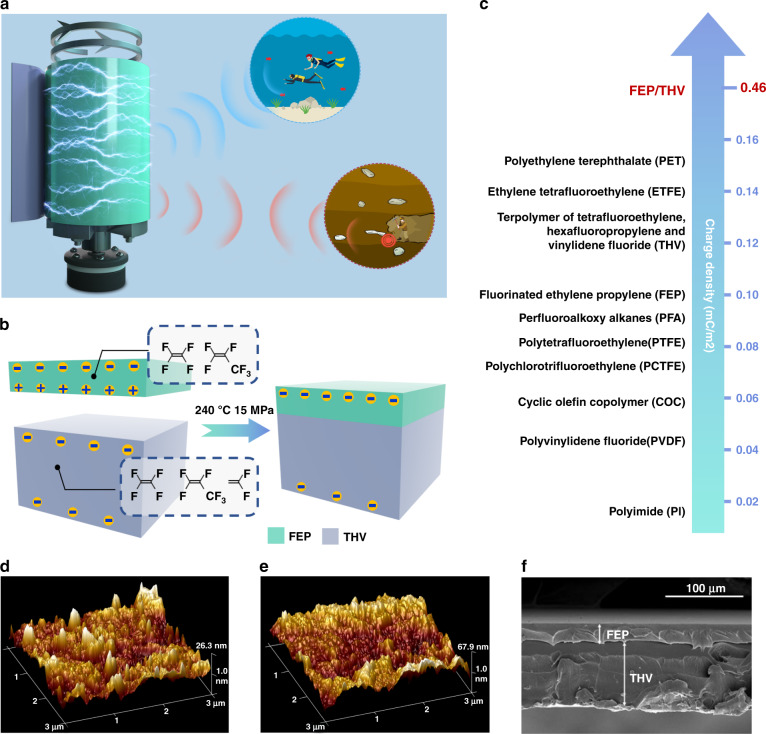
FEP/THV unipolar electret. **a** Principle and application of the EBMA based on FEP/THV unipolar electrets. **b** Fabrication of the FEP/THV electret by a hot-pressing process. **c** Electret series based on net charge density. AFM images of the **d** FEP surface and **e** THV surface of the FEP/THV electret. **f** Cross-sectional SEM image of the FEP/THV electret

Herein, we propose an EBMA by assembling a fluorinated ethylene propylene/terpolymer of tetrafluoroethylene, hexafluoropropylene and vinylidene fluoride (FEP/THV) unipolar electret with a mechanical rotator for potential ELF communications in air, underwater, and underground, as illustrated in Fig. 1a. The FEP/THV unipolar electret can store negative charges on both surfaces. Compared with traditional bipolar electrets and unipolar electrets with one metallized surface, the FEP/THV unipolar electret has a higher total charge density. In addition, simulations and experiments on multilayer electrets provide a new method for improving the performance of the EBMA. With the total charge density of a single-layer FEP/THV unipolar electret reaching −0.46 mC/m^2^, an EBMA based on an FEP/THV unipolar electret achieves several important features: (i) the transmitting efficiency of the EBMA based on an FEP/THV unipolar electret is significantly enhanced when compared to an EBMA with traditional bipolar or unipolar FEP electrets; (ii) for a 3-layer-FEP/THV-based EBMA with a compact volume of 5 × 10^−4^ m^3^, the transmitting distances in sea water, soil, and air can reach 71.4 m, 128.4 m, and 136.3 m, respectively, when the receiving magnetic flux density is 1 fT; (iii) with a power consumption < 5 W, binary ASCII coded ELF information of “BUAA” is successfully transmitted. This design strategy, together with the characteristics of the mechanical antenna in this work, provides the possibility for improving miniaturized ELF communications.

## Results and discussion

### Fabrication of FEP/THV unipolar electret

Traditional polymer FEP electrets have high negative charge storage stability^27–29^. However, without special treatment, the two FEP electret surfaces will carry charges of opposite polarity after corona charging^30^. Coating one surface of the FEP electret with an electrode can make it unipolar, but the metallized surface does not contribute to charge storage. Under certain conditions, some polar electrets, such as a THV electret, can carry negative charges on both surfaces^31,32^. In this work, we combine FEP electrets with THV electrets to fabricate unipolar electrets with high negative charge densities on both surfaces (Fig. 1b). Pressing the FEP and THV films at 240 °C and 15 MPa for 5 min, we obtain the FEP/THV electret film. The detailed preparation process is described in the Methods section. The polarization device is shown in Supplementary Fig. S1. Detailed polarization conditions are described in the Methods section. Compared with various unipolar electrets with one metallized surface, the FEP/THV electret has obvious advantages in net charge density (Fig. 1c), with a total charge density reaching −0.46 mC/m^2^ for one layer of electret.

Atomic force microscopy (AFM) images of the two FEP/THV surfaces are presented in Fig. 1d, e. The average roughness of the FEP and THV surfaces is 6.9 nm and 19.9 nm, respectively. The high roughness of the two surfaces is beneficial for charging storage of the electret. Scanning electron microscopy (SEM) images of the FEP/THV electret are presented in Fig. 1f. The FEP layer and THV layer are tightly combined after the hot-pressing process. The thickness of the FEP layer is approximately 30 μm, and the thickness of the THV layer is approximately 90 μm. The large thickness of the FEP/THV electret reduces the mutual influence between the negative charges on the two surfaces.

### Unipolar phenomenon of the FEP/THV electret

**Fig. 2:**
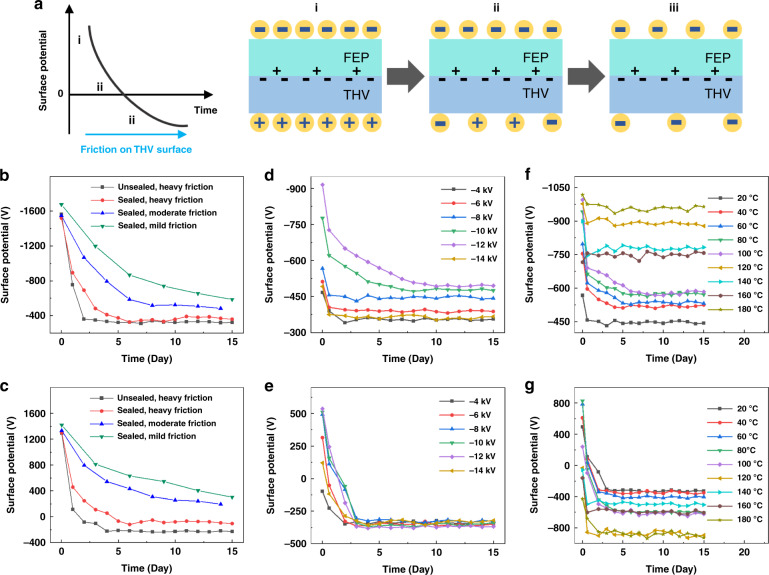
Investigating the mechanism and polarization of FEP/THV unipolar electrets. **a** The process of polarity reversal of the FEP/THV electret. The surface potential of both surfaces of the FEP/THV electret with different **b**, **c** storage conditions, **d**, **e** polarization voltages and **f**, **g** polarization temperatures. **b**, **d**, **f** FEP surface, **c**, **e**, **g** THV surface

The unipolar phenomenon of the FEP/THV electret does not appear immediately after polarization. The process of the polarity change on the two surfaces of the FEP/THV electret is shown in Fig. 2a. After corona polarization, due to the electrostatic induction effect, the two surfaces of the FEP/THV electret store charges with opposite polarities and similar amounts. The FEP surface (polarization surface) exhibits a negative potential with the same polarity as the polarization voltage, and the THV surface (nonpolarization surface) exhibits a surface positive potential. According to the measurement results of short-circuit thermally stimulated discharge (Supplementary Fig. S2), there are positive and negative charges at the interface between the FEP and THV layers, and the number of negative charges is approximately twice that of positive charges. With the passage of time, the negative charges injected by the corona polarization on the FEP surface and the positive charges generated by the electrostatic induction effect on the THV surface gradually decrease. The electrostatic induction effect is also gradually weakened. Meanwhile, the contact and friction with the external environment make the THV surface gain negative charges. As the negative charges obtained by friction gradually increase, exceeding the positive charges generated by the electrostatic induction effect, the potential of the THV surface changes from positive polarity to negative polarity, and finally, both surfaces of the FEP/THV electret have a negative potential to be a unipolar electret without a metallized surface.

Figure 2b,c compare the surface potentials of FEP/THV electrets with different friction situations. The polarization conditions of all electrets are the same (−8 kV, 20 °C, 10 min). We change the contact and friction between the THV surface and the external environment by changing the storage condition and measurement frequency. The higher the frequency of measurement is, the higher the frequency of friction, and the stronger the degree of friction. An unsealed FEP/THV electret placed in an open lab environment has the fastest charge decay speed and the shortest time to reach a steady state. Its THV surface reversed its polarity within two days after polarization. For a sealed electret stored in a sealed bag, the less the contact and friction with the external environment, the slower is the charge decay speed, and the longer is the time required for the polarity reversal of the THV surface. The FEP/THV electret with heavy friction has a polarity reversal on the THV surface within five days after polarization. The FEP/THV electrets with moderate friction and mild friction still have no polarity reversal within 15 days after polarization. Based on the experimental results, we can infer that if the FEP/THV electret does not have contact and friction with the external environment, its two surfaces will always exhibit opposite polarities. This proves that it is indeed the friction that gives the electret a unipolar phenomenon in which both surfaces are negative.

Figure 2d,e compare the surface potential of the FEP/THV electret with different polarization voltages (other polarization conditions except voltage are the same, 20 °C, 10 min). When the polarization voltage is higher than the breakdown limit of the electret (under the experimental conditions, the breakdown voltage is −14 kV), the electret is broken down and loses the ability to store charges, resulting in a low surface potential. Within the breakdown limit, the higher the polarization voltage is, the higher the potential on both surfaces of the electret after polarization, the longer the time for the electret potential to reach a steady state, and the higher the potential on the FEP surface in a stable state. For the THV surface, the high polarization voltage gives the surface a high initial potential. It takes a long time for the polarity reversal to occur. However, the potential difference in the steady state with different polarization voltages is small. According to the experimental results, −12 kV is the best polarization voltage. With a −12 kV polarization voltage, the two surfaces of the FEP/THV electret have the highest negative potential, which are ~ −495 V and ~ −370 V for the FEP and THV surfaces, respectively.

Figure 2f,g compare the surface potential of the FEP/THV electret with different polarization temperatures (other polarization conditions except temperature are the same, −8 kV, 10 min). We polarize the FEP/THV electret at 20 °C to 180 °C (the polarization conditions, such as voltage, are the same). Thus, 180 °C is the highest working temperature of the material, and if it exceeds 180 °C, the FEP layer and THV layer will be separated. When the polarization temperature is less than 120 °C, the surface potential of the FEP/THV electret increases with increasing polarization temperature. When the polarization temperature is between 120 and 180 °C, the initial potential of the two surfaces of the FEP/THV electret is negative, and as the polarization temperature increases, the negative potential on the FEP surface gradually decreases, and the negative potential on the THV surface gradually increases. When the polarization temperature is 180 °C, the potential on both surfaces of the FEP/THV electret increases again, and it is the maximum surface potential at each temperature. Therefore, 180 °C is the best polarization temperature for the FEP/THV electret. The surface potentials of the FEP and THV surfaces are ~ −910 V and ~ −860 V, respectively.

With the best polarization conditions (−12 kV, 180 °C, 10 min), the surface potential for the FEP and THV surfaces is ~ −970 V and ~ −920 V, respectively. According to Supplementary Fig. S3, the total charge density of the FEP/THV electret is ~ −0.46 mC/m^2^.

### Design optimization for the FEP/THV unipolar electret and EBMA

**Fig. 3:**
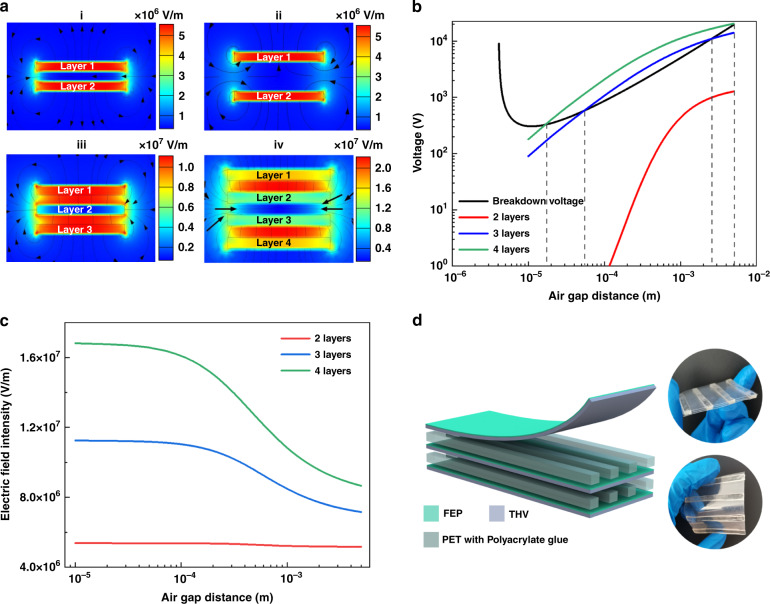
Design optimization for FEP/THV unipolar electrets. **a** COMSOL simulation of the electric field distribution of multilayer FEP/THV electrets with different air gap distances: (i) 2 layers, air gap distance is 100 μm; (ii) 2 layers, air gap distance is 300 μm; (iii) 3 layers, air gap distance is 100 μm; (iv) 4 layers, air gap distance is 100 μm. The charge density on both surfaces of each electret is set to the value measured by the experiment. **b** The relationship between the voltage drops inside the multilayer FEP/THV electret air gap and the air gap distance. **c** Relationship between the electric field intensity inside the electret and the air gap distance between the multilayer FEP/THV electret. **d** Schematic diagram and photos indicating the structure of the 3-layer FEP/THV electret

Except for increasing the net charge density of single-layer electrets, multilayer FEP/THV electrets can enable EBMAs with more charges, thereby improving radiation performance. The number of layers of the multilayer electret and the air gap distance between every two layers will affect the electric field distribution near the electret **(**Fig. 3a**)**. When the electric field is too strong, the air gap will be broken down, which will reduce the performance of the EMBA.

The breakdown of the air gap between the multilayer electret conforms to Paschen’s law (Eq. 1). When the air pressure is constant, the breakdown voltage (*V*_*bre*_) is related to the air gap distance (*d*)^33–35^.

1 $$V_{{{{\mathrm{bre}}}}} = \frac{{APd}}{{\ln \left( {BPd} \right) - \ln \left[ {\ln \left( {1 + \frac{1}{{\gamma _{{{{\mathrm{se}}}}}}}} \right)} \right]}}$$

where *A* is related to the excitation and ionization energies, *B* is the saturation ionization in the gas at a particular ratio of electric field intensity to pressure, *P* is the gas pressure, and *γ*_se_ is the secondary-electron-emission coefficient.

For the 2-layer FEP/THV electret, no matter how long the air gap is, no air will be broken down (Fig. 3b). For the 3-layer FEP/THV electret, when the air gap distance is less than 56 μm or greater than 2.6 mm, the voltage between each layer electret is lower than the breakdown voltage described by Paschen’s law. For the 4-layer FEP/THV electret, the appropriate air gap distance is less than 1.8 μm or greater than 5.1 mm. Therefore, we can always find a suitable air gap distance so that the air between the electrets will not be broken down by the high electrical field generated by the charges carried by the electret.

As the number of layers increases, the electric field strength inside the electret increases (Fig. 3c). The smaller the air gap distance is, the greater the electric field strength. In addition, since the surfaces of each layer’s electret carry charges of the same polarity, the charges will affect each other at close distances. Therefore, a large air gap distance is more advantageous. For example, for a 3-layer FEP/THV electret, we choose an air gap distance greater than 2.6 mm. To maintain a certain air gap distance and fix the multilayer electret, we use a 3 mm thick PET with polyacrylate glue as the spacer and adhesive layer to prepare the multilayer FEP/THV electret. A schematic diagram and photographs showing the structure of the 3-layer FEP/THV electret are provided in Fig. 3d. Specifically, the distance between each spacer on the same layer is 1.5 cm, and the length of each spacer is 6 cm.

**Fig. 4:**
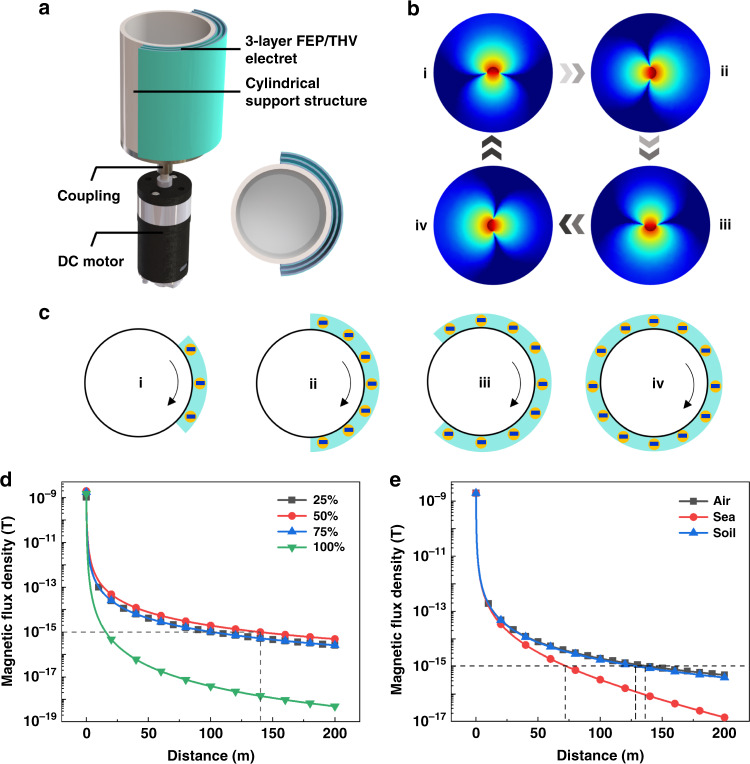
Design optimization for EBMA. **a** The structure of the 3-layer-FEP/THV-based EBMA. **b** COMSOL simulation of the magnetic field distribution of the rotating EBMA. The electret occupies 50% of the cylinder, and the rotation angles are (i) 0°, (ii) 90°, (iii) 180°, and (iv) 270°. **c** The covering area of the electret on the cylinder: (i) 25%; (ii) 50%; (iii) 75%; (iv) 100%. **d** COMSOL simulation of the magnetic flux density of EBMA with different electret coverage areas. **e** Magnetic flux density attenuation with distance in different media of air, sea water, and soil

Attaching the electret to the cylindrical structure and using a motor to drive the electret for rotation is a feasible EBMA solution. As shown in Fig. 4a, the 3-layer FEP/THV electret is covered on the outside of a cylinder with a radius of 4 cm and a height of 10 cm. The cylinder is connected to the DC motor through coupling. When the DC motor drives the electret to rotate, EBMA generates a magnetic signal with a specific frequency. A detailed COMSOL electromagnetic field simulation of the EBMA is described in the Methods section. In the actual simulation process, we take the π/360 rotation angle as the simulation step size to obtain the dynamic simulation result (Supplementary Video 1), and 4 typical static states are used to represent the process of changing the field during the rotation. When the electret rotates with the cylinder, the distribution of the magnetic field and electric field generated by the electret also periodically changes (Fig. 4b and Supplementary Fig. S4). In the axis direction of the EBMA, the magnetic field does not change and is a constant magnetic field. In the radial direction of the EBMA, the magnetic flux density and direction of the magnetic field change periodically, which means that the EBMA generates a magnetic field signal with the same frequency as the rotation frequency.

The distribution of the electret on the cylinder will affect the performance of the EBMA. When the electret covers different area proportions of the cylinder (Fig. 4c), the magnetic flux density generated by the 3-layer-FEP/THV-based EBMA is shown in Fig. 4d (the charge density of each FEP/THV electret is −0.46 mC/m^2^, the rotation frequency of the EBMA is 22.5 Hz). When the electret covers less than 50% of the area of the cylinder, the magnetic fields generated by the charges at each position on the cylinder are superimposed on each other. When the area of the electret is greater than 50%, the electric charges at the symmetrical position about the center of the circle have different moving directions, and the magnetic fields generated at the position outside the cylinder cancel each other. Therefore, when the electret area is 50%, the magnetic flux density generated by the EBMA is the largest.

With the best design optimization, the magnetic field propagation of the 3-layer-FEP/THV-based EBMA in air, sea water, and soil is shown in Fig. 4e (the conductivity of sea water is 4 S/m, the conductivity of soil is 0.015 S/m). When the receiving magnetic flux density is 1 fT^36–41^, the effective working distance of the antenna is 136.3 m in the air, 71.4 m in the sea water, and 128.4 m in the soil. It should be noted that magnetic field sensors, including superconducting quantum interference devices, SERF atomic magnetometers, and optical magnetometry, can achieve fT-level and sub-fT measurements.

### Test and demonstration of EBMA

**Fig. 5:**
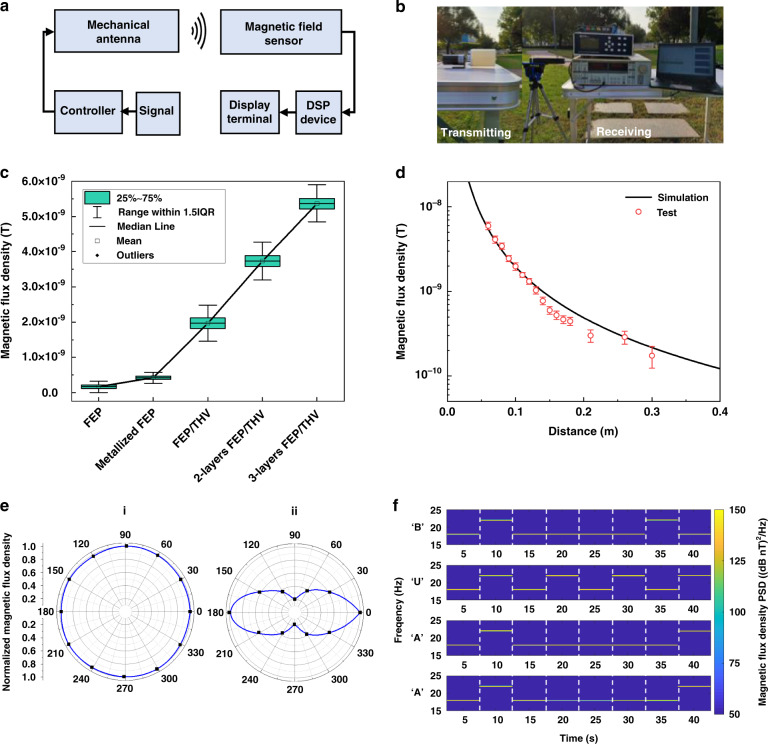
Test and demonstration of EBMA. **a** EBMA signal transceiver system design. **b** Photo showing the real test scenario of EBMA. **c** Performance comparison of EBMA using different electrets of bipolar FEP, unipolar FEP with one metallized surface, and 1-, 2-, 3-layer FEP/THV unipolar electret. **d** Simulated and tested magnetic flux density versus distance and **e** radiation pattern of a 3-layer FEP/THV-based EBMA. **f** Transmitting “BUAA” with the FSK communication method by a 3-layer FEP/THV-based EBMA

The EBMA signal transceiver system consists of two parts: transmitting and receiving components (Fig. 5a). The data signal to be sent is processed by the controller and converted into an electrical signal, which drives the EBMA to produce a movement with the corresponding frequency. Then, the EBMA emits a coded magnetic signal. The magnetic field sensor receives the magnetic signal emitted by the EBMA. The magnetic signal is processed by the digital signal process (DSP) device and then transmitted to the terminal, where it is demodulated into the original signal. The real test scenario is shown in Fig. 5b. The detailed EBMA prototype production and test process is described in the Methods section.

By changing the electret material used for the EBMA, the intensity of radiation produced by the EBMA changes. The magnetic flux density is positively correlated with the net charge density of the electret. At the same receiving distance (8 cm), the magnetic flux density generated by the EBMA using the FEP/THV electret is 4.63 times that of the FEP electret with one metallized surface and 11.66 times that of the bipolar FEP electret (Fig. 5c). Tests on the magnetic flux density of the EBMA using different layers of FEP/THV electrets show that increasing the number of electret layers increases the magnetic flux density approximately linearly. The peak magnetic flux density generated by a 3-layer-FEP/THV-based EBMA is ~ 5.36 nT.

Measuring the magnetic flux density generated by a 3-layer-FEP/THV-based EBMA at different distances, the measurement results are consistent with the aforementioned simulation results (Fig. 5d). At a distance of 0.3 m, the magnetic flux density produced by the 3-layer-FEP/THV-based EBMA is approximately 0.1 nT. By increasing the number of electret layers, increasing the size, or using a higher-precision magnetic field sensor, the FEP/THV-based EBMA can achieve a longer effective working distance. Within the measurement distance, the attenuation of the magnetic flux density generated by the EBMA with the distance (r) is $\frac{1}{{r^2}}$. The theoretical model provided in Supplementary Fig. S5 also supports this conclusion. This is one of the advantages of an EBMA compared to a magnet-based mechanical antenna (MBMA), since the attenuation of the magnetic flux density generated by an MBMA with the distance is $\frac{1}{{r^3}}$ ^42–44^. An EBMA has a small attenuation in the near field. Moreover, when the intensity of the radiation source is the same, the working distance of the EBMA is farther than that of the MBMA. For another kind of mechanical antenna based on piezoelectric materials, its work frequency is normally above very low frequency (3 kHz ~ 30 kHz), and its application scenarios are different from the EBMA proposed in this work.

The magnetic flux density was measured with the corresponding signal frequency at each position in the EBMA rotating plane and in the plane perpendicular to the rotating plane at the same distance (8 cm), as shown in Fig. 5e. In the rotating plane, the magnetic flux density is equal at each position at the same distance from the EBMA. The EBMA is an omnidirectional antenna in a rotating plane. In the plane perpendicular to the rotating plane, the magnetic flux density in the direction of the EBMA axis is the smallest, and the magnetic flux density in the direction perpendicular to the axis is the largest.

For the EBMA, direct antenna modulation (DAM) is a suitable modulation method. By directly changing the rotation frequency of the EBMA to change the frequency of the magnetic signal, using frequency shift keying (FSK), signals with different frequencies represent different data information (Supplementary Fig. S6). The maximum frequency of FSK and the frequency bandwidth that can express information depend on the mechanical properties such as the torque and speed of the motor. We use 0.1 Hz FSK and expressed signals of ‘0’ and ‘1’ with 17.5 Hz and 22.5 Hz, then conduct EBMA signal transmission and reception tests. The distance between the signal receiving device and the EBMA is 8 cm. Under power for an EBMA less than 5 W, the ELF information of “BUAA” transmitted by the EBMA using binary ASCII code is clearly presented on the receiving terminal (Fig. 5f), demonstrating one application example of our proposed EBMA. It should be noted that the transmitting distance can be extended when the size/power of the EBMA are increased or when a magnetic sensor with higher sensitivity is used.

## Conclusion

In this work, an electret-based mechanical antenna with high radiation performance using FEP/THV unipolar electrets is proposed. We confirm that the unipolarity of the FEP/THV electret is caused by friction and utilize the unipolarity of the FEP/THV electret to achieve a total charge density of −0.46 mC/m^2^ for a single layer of electret. Furthermore, the stacking of multiple layers of FEP/THV electrets enables an EBMA to have stronger radiation performance. By optimizing the design of the electret distribution, the EBMA can spread 136.3 m in the air, 71.4 m in the sea water, and 128.4 m in the soil when the receiving magnetic flux density is 1 fT. As an application demonstration, ELF information of “BUAA” is transmitted with the FSK communication method by a 3-layer FEP/THV-based EBMA, with small power consumption (< 5 W). Our work improves the performance of an EBMA and provides an effective strategy for developing miniaturized antennas for air, underwater, and underground ELF communications.

## Methods

### Fabrication

The FEP/THV film is fabricated by hot pressing. Two grams of THV815 pellets was placed into a flat vulcanizer (ST15-YP, Kunshan Lugong Precision Instrument Co., Ltd.) kept at 240 °C for 2 min to allow the THV815 pellets to melt completely. The mixture was pressurized to 10 MPa and maintained for 5 min. After cooling, the THV film was removed. Then, the THV film and the commercial FEP film were placed into a flat vulcanizer, the temperature was set to 240 °C, the pressure was set to 15 MPa, and the temperature was maintained for 5 min. After cooling, the FEP/THV film was removed.

### Polarization

The electret is polarized by corona polarization. A constant voltage grid-controlled corona polarization device generates a uniform electric field (Supplementary Fig. 2). For default conditions, the polarization voltage is −8 kV, the polarization temperature is 20 °C, and the polarization time is 10 min. When a certain polarization condition is changed, the other conditions remain the same.

### COMSOL electromagnetic field simulation

A cylinder with a radius of 4 cm and a height of 10 cm is established as the geometric model of an EBMA. An electret attached to the cylinder is simulated by loading an electric charge on the outer wall of the cylinder. The surface charge density is set according to the total charge density of the electret we use. With the moving mesh module, the central axis of the cylinder is set as the rotation axis, and then the rotation frequency is set to 22.5 Hz. The transient electromagnetic wave module is used to solve Maxwell’s equations for finite element simulation of the EBMA’s magnetic field radiation characteristics.

### EBMA prototype production

The EBMA is composed of a DC motor, a coupling, a supporting structure and an electret. The DC motor is a JGB37-3650 brushless DC gear motor. The maximum rotation frequency of the DC motor is 22.5 Hz, and the torque at this frequency is 0.12 N·m. The coupling is a 6 mm D hole M2006 motor coupling. The supporting structure is a thin-walled cylinder made of acrylonitrile butadiene styrene (ABS) plastic. The radius of the cylinder is 4 cm, and the length is 10 cm. The 3-layer FEP/THV electret occupies 50% of the area of the cylinder.

### Radiation performance tests

The magnetic field sensor that receives the magnetic signal is a Bartington Mag-13 fluxgate. The DSP device is a Stanford Research Systems SR810 lock-in amplifier. When measuring the magnetic flux density generated by the EBMA using different electrets, the relationship between magnetic flux density and distance, and FSK modulation, the receiving device is located in the plane of the vertical line of the cylinder axis. The total measured magnetic field is generated by the motor and the electret, and the two sources have the same phase and frequency. At each test point, we first measure the magnetic field generated by the motor without an electret, which is the background value. The magnetic field generated by the electret is the difference between the total measured value and the background value.

## Supplementary information

Supplementary Materials

Supplementary Video 1

## Data Availability

All relevant data are available from the corresponding author upon request.
