# Supplementary material for: A miniaturized mechanical antenna based on FEP/THV unipolar electrets for extremely low frequency transmission
Source: Microsyst Nanoeng. 2022 May 31;8:58. doi: 10.1038/s41378-022-00395-x (PMC9151712; doi:10.1038/s41378-022-00395-x)
Supplement: Supplementary file 1 — Supplementary Materials [file 41378_2022_395_MOESM1_ESM.docx]

*Supplementary Information for*

A Miniaturized Mechanical Antenna Bases on FEP/THV Unipolar Electret for Extremely-Low Frequency Transmission

*Yong Cui^1*^, Ming Wu^1^, Zhaoyang Li^2^, Xiao Song^3*^, Chen Wang^1^, Haiwen Yuan^1^, Zhi-Xin Yang^4^, Junwen Zhong^2*^*

^1^ School of Automation Science and Electrical Engineering, Beihang University, Beijing 100191, China.

^2^ Department of Electromechanical Engineering and Centre for Artificial Intelligence and Robotics, University of Macau, Macau SAR, 999078, China.

^3^ School of Cyber Science and Technology, Beihang university, Beijing 100191, China

^4^ State Key Laboratory of Internet of Things for Smart City and Department of Electromechanical Engineering, University of Macau, Macau SAR, 999078, China.

*Corresponding author: Yong Cui, Xiao Song, Junwen Zhong

[cuiyong@buaa.edu.cn](mailto:cuiyong@buaa.edu.cn)

[songxiao@buaa.edu.cn](mailto:songxiao@buaa.edu.cn)

[junwenzhong@um.edu.mo](mailto:junwenzhong@um.edu.mo)


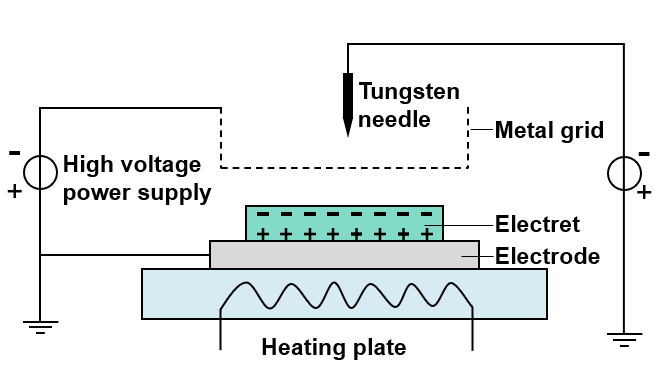


**Supplementary Fig. S1.** Corona polarization device.


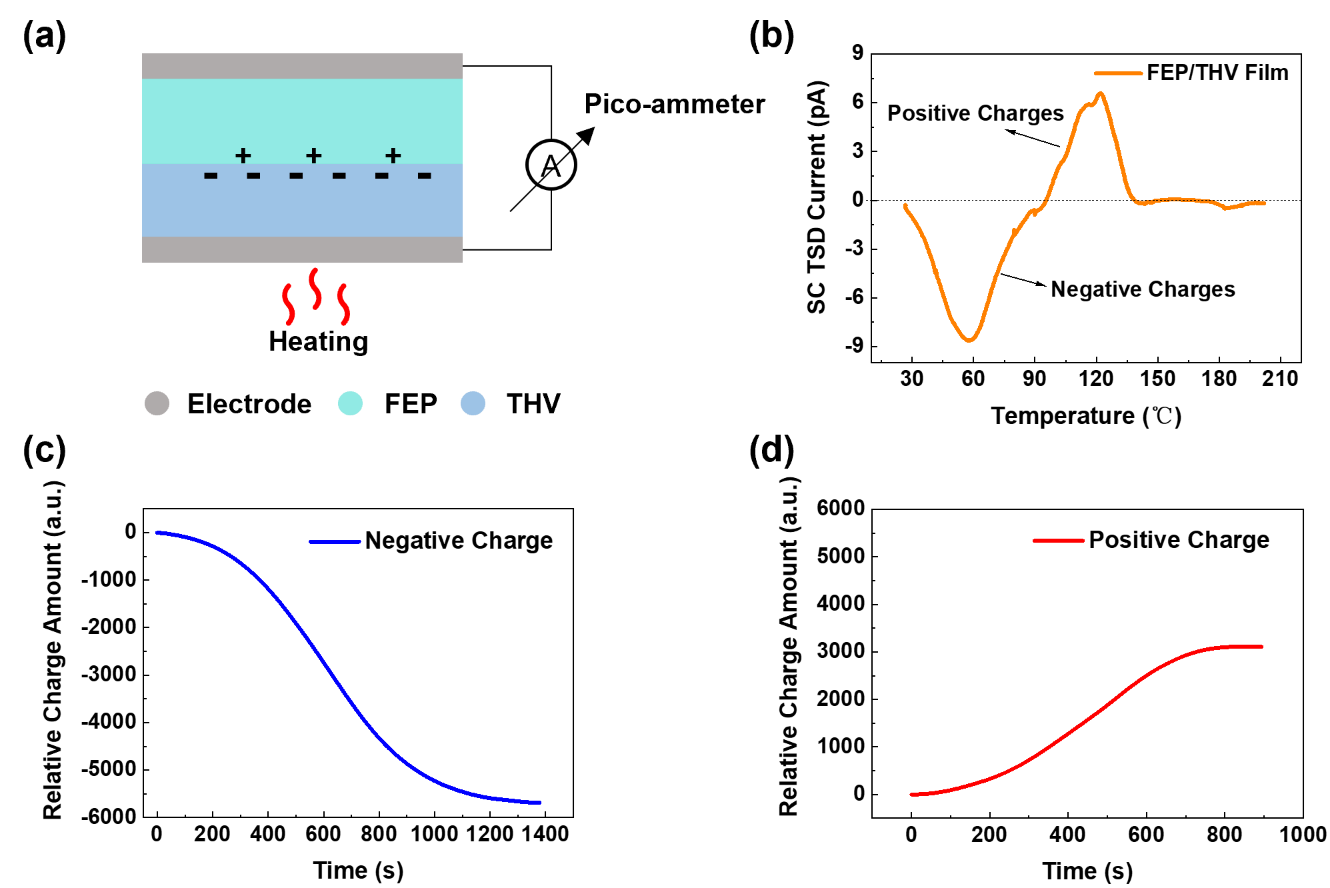


**Supplementary Fig. S2.** Short-circuit thermally stimulated discharge (SC TSD) measurement results of FEP/THV electret. (a) SC TSD model. (b) SC TSD current. (c) The amount of negative charges at the FEP/THV electret interface. (d) The amount of positive charges at the FEP/THV electret interface.


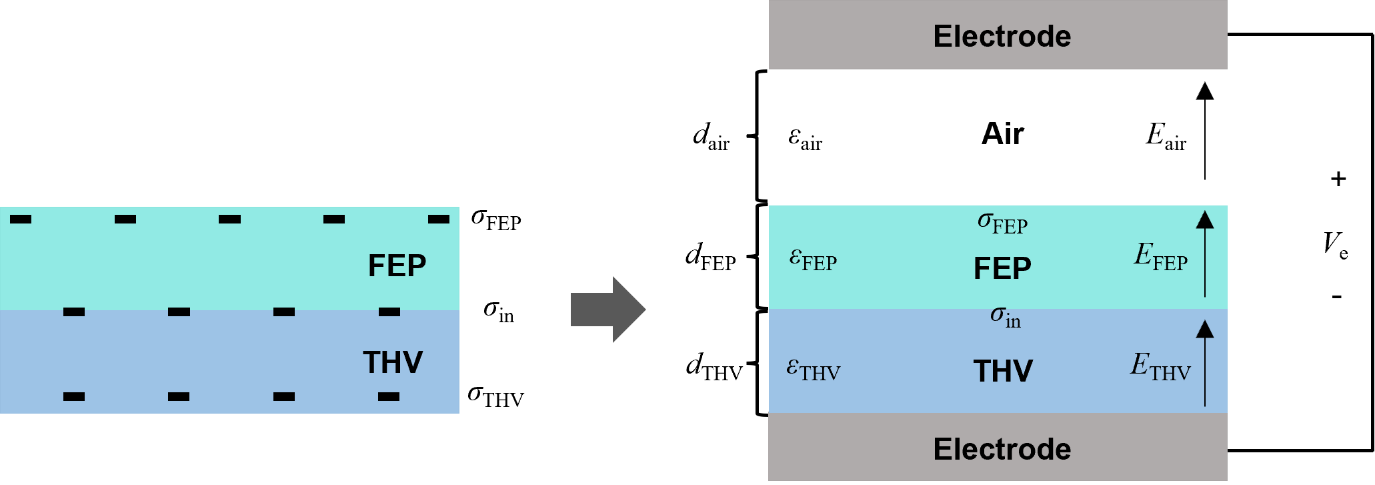


**Supplementary Fig. S3.** Charge density calculation model of FEP/THV electret.

We can approximate that FEP/THV electret has three layers of charges on the surface and interface, and the charge density calculation model is shown in **Supplementary Fig. S3**. According to Gauss's law, the electric displacement equation of each surface and interface is as follows:

$\left\{\begin{matrix} \varepsilon_{\text{air}}E_{\text{air}}-\varepsilon_{\text{FEP}}E_{\text{FEP}}=\frac{\sigma_{\text{FEP}}}{\varepsilon_{0}} \\ \varepsilon_{\text{FEP}}E_{\text{FEP}}-\varepsilon_{\text{THV}}E_{\text{THV}}=\frac{\sigma_{\text{in}}}{\varepsilon_{0}} \end{matrix}\right.$ (1)

where, $\varepsilon_{\text{air}}$, $\varepsilon_{\text{FEP}}$ and $\varepsilon_{\text{THV}}$ are the relative permittivity of air (~1), FEP (~2.1) and THV(~4). $\varepsilon_{0}$ is the vacuum dielectric constant. $E_{\text{air}}$, $E_{\text{FEP}}$ and $E_{\text{THV}}$ are the internal electric field strength of air, FEP and THV. When measuring surface potential, $E_{\text{air}}$ is equal to 0. $\sigma_{\text{FEP}}$ is the charge density on FEP surface. $\sigma_{\text{in}}$ is the charge density at the interface between FEP and THV.

According to Kirchhoff's second law:

$d_{\text{air}}E_{\text{air}}+d_{\text{FEP}}E_{\text{FEP}}+d_{\text{THV}}E_{\text{THV}}+V_{\text{e}}=0$ (2)

where, $d_{\text{air}}$, $d_{\text{FEP}}$ and $d_{\text{THV}}$ are the thickness of air (~1 cm), FEP (~30 μm) and THV (~90 μm). $V_{\text{e}}$ is the voltage of the external circuit, that is, the surface potential.

$\sigma_{FEP}$ and $\sigma_{THV}$ are the charge densities of FEP and THV electrets with one metallized surface with the same polarization conditions. The thickness of electret with one metallized surface is the same as that of FEP/THV electret (~120 μm).

Solve the equation (1) and (2) to get $\sigma_{in}$, then the total charge density $\sigma_{total}$ of FEP/THV electret can be obtained.

$\sigma_{total}=\sigma_{FEP}+\sigma_{THV}+\sigma_{in}$ (3)


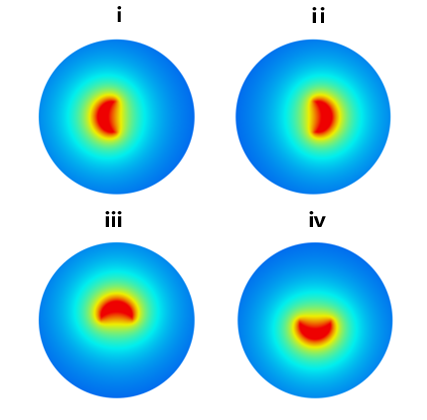


**Supplementary Fig. S4.** Electric field distribution of rotating EBMA, the electret occupies 50% of the cylinder, rotation angle of (ⅰ) 0°; (ⅱ) 90°; (ⅲ) 180°; (ⅳ) 270°.

The mechanism of electromagnetic field radiation generated by rotating FEP/THV based EBMA can be equivalent to electromagnetic field radiation generated by a charged particle moving in a uniform circular motion:


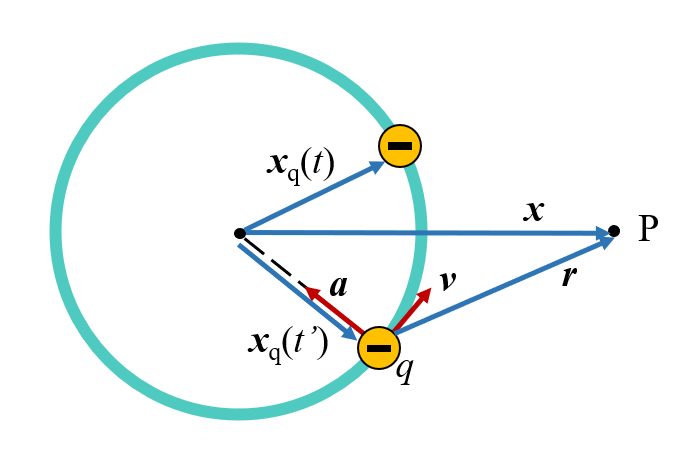


**Supplementary Fig. S5.** Equivalent radiation model of rotating FEP/THV based EBMA.

Suppose the amount of charge carried by a charged particle is *q*:

$q=n\rho_{\text{ele}}S$ (4)

where, *n* is the number of electret layers, $\rho_{\text{ele}}$ is the charge density of FEP/THV electret, *S* is the area of the electret.

According to the delay effect, the electromagnetic scalar potential and electromagnetic vector potential $A\left(x,t\right)$ generated by the charge *q* moving along a certain trajectory at time *t* to the point P at *x* depend on the charge density distribution $\rho\left(x_{\text{q}}\left(t'\right),t'\right)$ and current density distribution $j\left(x_{\text{q}}\left(t'\right),t'\right)$ of the charge at the time of delay $t'$:

(5)

where, $x_{\text{q}}\left(t'\right)$ is the position vector of the charge in time $t'$, $x_{\text{q}}\left(t\right)$ is the position vector of the charge in time $t$, $\varepsilon_{0}$ is the vacuum dielectric constant, *r* is the modulus of the vector ***r*** pointing to the point P at the position of the charge at time $t'$, $\mu_{0}$ is the vacuum permeability.

The relationship between the delay time $t'$ and the initial time t is:

$t'=t-\frac{r}{c}$ (6)

where, c is the speed of light.

In the process of charge movement, for any time *t*, there is only a specific delayed time $t'$, when the electromagnetic signal sent out can reach the field point P at time *t*.

Solving Equation (5) electromagnetic potential integral, the results are as follows:

(7)

$A\left(x,t\right)=\frac{\mu_{0}n\rho_{\text{ele}}Sv}{4\text{π}\left(r-\frac{1}{c}v\cdot r\right)}$ (8)

where, ***v*** is the velocity vector of the charge in time $t'$.

Since the electric charge moving speed *v* is much smaller than the speed of light *c* during the working process of the EBMA, the Equation (7) and Equation (8) can be rewritten approximately as:

(9)

$A\left(x,t\right)=\frac{n\rho_{\text{ele}}S}{4\text{π}\varepsilon_{0}c^{2}r}$ (10)

The conditions set by the foregoing:

$r=\left|x-x_{\text{q}}\left(t'\right)\right|=c\left(t-t'\right)$ (11)

Therefore：

$\frac{\partial r}{\partial t'}=-\frac{v\cdot r}{r}=-v\cdot\hat{r}$ (12)

where, $\hat{r}$ is the unit vector in direction $r$.

$\frac{\partial t'}{\partial t}=1-\frac{1}{c}\frac{\partial r}{\partial t'}\frac{\partial t'}{\partial t}=1+\frac{v\cdot\hat{r}}{c}\frac{\partial t'}{\partial t}$ (13)

$\frac{\partial t'}{\partial t}=\frac{1}{1-\frac{v\cdot\hat{r}}{c}}$ (14)

Since $v\ll c$, so：

$\frac{\partial t'}{\partial t}\approx1$ (15)

$\nabla t'=-\frac{1}{c}\nabla r=\left.-\frac{1}{c}\nabla r\right|_{t'\text{constant}}-\frac{1}{c}\frac{\partial r}{\partial t'}\nabla t'=-\frac{\hat{r}}{c}+\frac{v\cdot\hat{r}}{c}\nabla t'$ (16)

Therefore：

$\nabla t'=-\frac{\hat{r}}{c\left(1-\frac{v\cdot\hat{r}}{c}\right)}\approx-\frac{\hat{r}}{c}$ (17)

Based on the above results, the magnetic induction intensity generated by the moving charge is as follows:

$\begin{matrix} B=\nabla\times A \\ =\left.\nabla\times A\right|_{t'\text{constant}}+\nabla t'\times\frac{\partial A}{\partial t'} \\ =\frac{1}{4\text{π}\varepsilon_{0}c^{2}}\nabla\frac{1}{r}\times v+\nabla t'\times\frac{\partial A}{\partial t'} \\ =\frac{n\rho_{\text{ele}}Sv\times\hat{r}}{4\text{π}\varepsilon_{0}c^{2}r^{2}}+\frac{\hat{r}}{c}\times\frac{n\rho_{\text{ele}}Sv\left(v\cdot\hat{r}\right)}{4\text{π}\varepsilon_{0}c^{2}r^{2}}+\frac{\hat{r}}{c}\times\frac{n\rho_{\text{ele}}Sa}{4\text{π}\varepsilon_{0}c^{2}r} \\ =\frac{n\rho_{\text{ele}}Sv\times\hat{r}}{4\text{π}\varepsilon_{0}c^{2}r^{2}}\left(1+\frac{v\cdot\hat{r}}{c}\right)+\frac{n\rho_{\text{ele}}Sa\times\hat{r}}{4\text{π}\varepsilon_{0}c^{3}r} \\ \approx\frac{n\rho_{\text{ele}}S}{4\text{π}\varepsilon_{0}}\left(\frac{v\times\hat{r}}{c^{2}r^{2}}+\frac{a\times\hat{r}}{c^{3}r}\right) \end{matrix}$ (18)

where, ***a*** is the acceleration of the moving charge.

In the same way, the electric field intensity generated by the moving charge can be obtained as follows:

(19)

Since the charge moves in a uniform circular motion, we can get:

$v=\omega R$ (20)

$a=\omega^{2}R$ (21)

where, $\omega$ is the rotational angular velocity, *R* is the radius of circular motion, that is, the radius of the cylindrical support structure.

Therefore, the electromagnetic field radiation model generated by the rotating FEP/THV based EBMA is:

$\left\{\begin{matrix} B=\frac{n\rho_{\text{ele}}S\omega R}{4\text{π}\varepsilon_{0}c^{2}r^{2}}\hat{v}\times\hat{r}+\frac{n\rho_{\text{ele}}S\omega^{2}R}{4\text{π}\varepsilon_{0}c^{3}r}\hat{a}\times\hat{r} \\ E=\frac{n\rho_{\text{ele}}S}{4\text{π}\varepsilon_{0}r^{3}}\hat{r}+\frac{n\rho_{\text{ele}}S\omega^{2}R}{4\text{π}\varepsilon_{0}c^{2}r}\hat{r}\times\left(\hat{r}\times\hat{a}\right) \end{matrix}\right.$ (22)

From Equation (22), the magnetic induction intensity generated by the rotating FEP/THV based EBMA is related to the number of electret layers, the charge density of each electret, the area of the electret and the cylindrical support structure. The radius is proportional, and the higher the antenna rotation frequency, the greater the magnetic induction intensity generated.

Since the second term of the expression of magnetic induction and electromagnetic intensity in Equation (22) is much smaller than the first term, it can be approximated that for a rotating unipolar electret mechanical antenna, the magnetic induction intensity generated by it decays inversely proportional to the square with the distance, and the electric field intensity decays inversely proportional to the cubic with the distance.


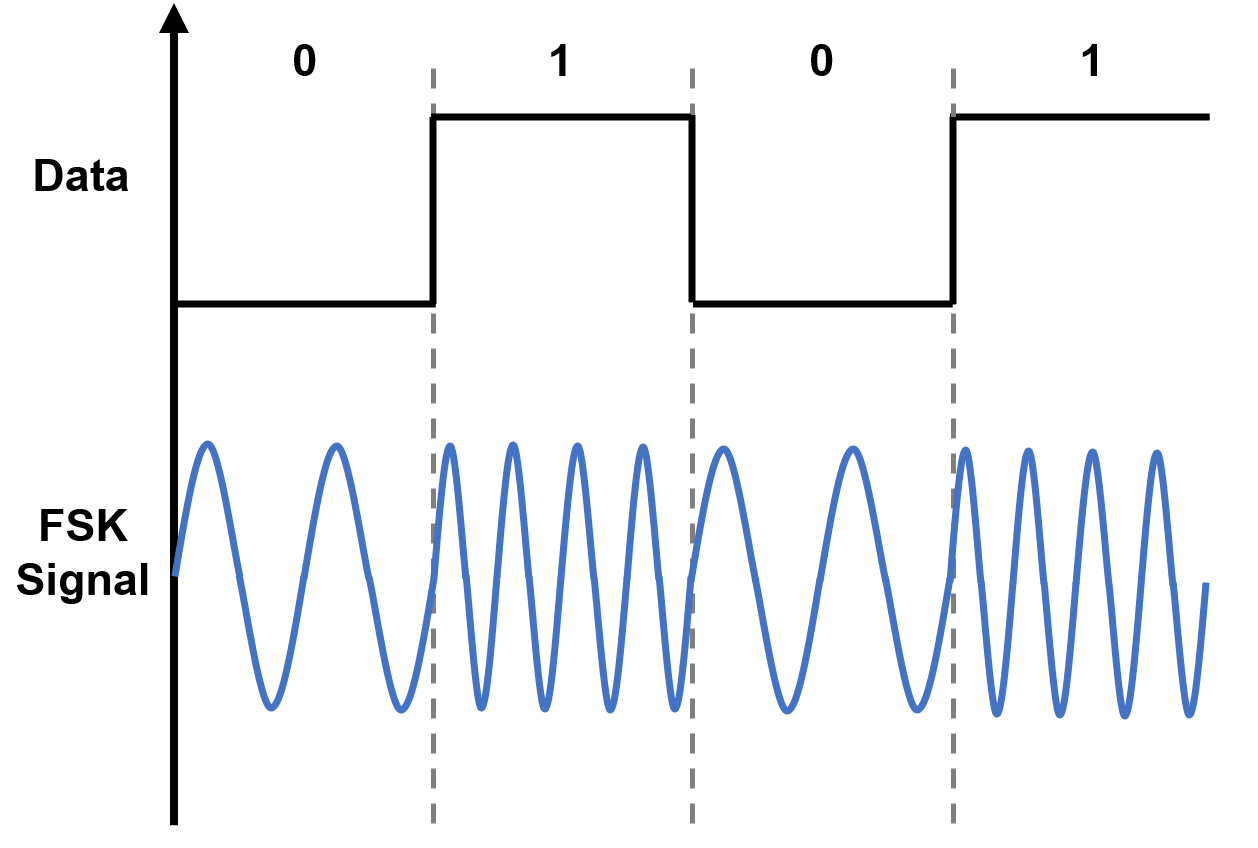


**Supplementary Fig. S6.** FSK signal modulation.
